# Artificial Intelligence–Assisted Endoscopic Diagnosis of Early Upper Gastrointestinal Cancer: A Systematic Review and Meta-Analysis

**DOI:** 10.3389/fonc.2022.855175

**Published:** 2022-06-10

**Authors:** De Luo, Fei Kuang, Juan Du, Mengjia Zhou, Xiangdong Liu, Xinchen Luo, Yong Tang, Bo Li, Song Su

**Affiliations:** ^1^ Department of Hepatobiliary Surgery, The Affiliated Hospital of Southwest Medical University, Luzhou, China; ^2^ Department of General Surgery, Changhai Hospital of The Second Military Medical University, Shanghai, China; ^3^ Department of Clinical Medicine, Southwest Medical University, Luzhou, China; ^4^ Department of Ultrasound, Seventh People’s Hospital of Shanghai University of Traditional Chinese Medicine, Shanghai, China; ^5^ Department of Hepatobiliary Surgery, Zigong Fourth People’s Hospital, Zigong, China; ^6^ Department of Gastroenterology, Zigong Third People’s Hospital, Zigong, China; ^7^ School of Computer Science and Engineering, University of Electronic Science and Technology of China, Chengdu, China

**Keywords:** artificial intelligence, upper gastrointestinal tract, early detection of cancer, endoscopy, systematic review

## Abstract

**Objective:**

The aim of this study was to assess the diagnostic ability of artificial intelligence (AI) in the detection of early upper gastrointestinal cancer (EUGIC) using endoscopic images.

**Methods:**

Databases were searched for studies on AI-assisted diagnosis of EUGIC using endoscopic images. The pooled area under the curve (AUC), sensitivity, specificity, positive likelihood ratio (PLR), negative likelihood ratio (NLR), and diagnostic odds ratio (DOR) with 95% confidence interval (CI) were calculated.

**Results:**

Overall, 34 studies were included in our final analysis. Among the 17 image-based studies investigating early esophageal cancer (EEC) detection, the pooled AUC, sensitivity, specificity, PLR, NLR, and DOR were 0.98, 0.95 (95% CI, 0.95–0.96), 0.95 (95% CI, 0.94–0.95), 10.76 (95% CI, 7.33–15.79), 0.07 (95% CI, 0.04–0.11), and 173.93 (95% CI, 81.79–369.83), respectively. Among the seven patient-based studies investigating EEC detection, the pooled AUC, sensitivity, specificity, PLR, NLR, and DOR were 0.98, 0.94 (95% CI, 0.91–0.96), 0.90 (95% CI, 0.88–0.92), 6.14 (95% CI, 2.06–18.30), 0.07 (95% CI, 0.04–0.11), and 69.13 (95% CI, 14.73–324.45), respectively. Among the 15 image-based studies investigating early gastric cancer (EGC) detection, the pooled AUC, sensitivity, specificity, PLR, NLR, and DOR were 0.94, 0.87 (95% CI, 0.87–0.88), 0.88 (95% CI, 0.87–0.88), 7.20 (95% CI, 4.32–12.00), 0.14 (95% CI, 0.09–0.23), and 48.77 (95% CI, 24.98–95.19), respectively.

**Conclusions:**

On the basis of our meta-analysis, AI exhibited high accuracy in diagnosis of EUGIC.

**Systematic Review Registration:**

https://www.crd.york.ac.uk/PROSPERO/, identifier PROSPERO (CRD42021270443).

## Introduction

Upper gastrointestinal cancer (UGIC) is among the most common malignancies and causes of cancer­related deaths worldwide, which presents a major challenge for health-care system (1). A majority of UGIC patients are detected at a late stage and have a poor prognosis. In contrast, with early detection, the 5-year overall survival can be more than 90% (2, 3). Thus, the early detection of UGIC is essential to improve patient prognosis.

Endoscopy remains the most optimal approach of UGIC detection (4, 5). However, endoscopic features of early upper gastrointestinal cancer (EUGIC) lesions are subtle and easily missed. Moreover, diagnostic accuracy depends on the expertise of endoscopists (2). One report revealed that EUGIC misdiagnosis can be high regardless of the number of patients, developed or underdeveloped locations, or in countries performing a remarkably high volume of endoscopies (6).

Artificial intelligence (AI) is gaining much popularity in the field of medicine, including gastrointestinal endoscopy (7–11). Owing to its good pattern recognition ability, AI is a promising candidate for detection of upper gastrointestinal lesions (12, 13). However, the data on AI-assisted EUGIC diagnosis are still lacking. Hence, we conducted this study to assess the diagnostic accuracy of AI in the detection of EUGIC using endoscopic images.

## Methods

This systematic review and meta-analysis was reported in line with PRISMA guidelines and was registered with the international prospective register of systematic reviews PROSPERO (CRD42021270443).

### Search Strategy and Study Selection

Two authors (FK and JD) separately searched electronic databases (PubMed, Medline, Embase, Web of Science, Cochrane library, and Google scholar) from the date of establishment until November 2021 using the following pre-specified search terms: “endoscopy”, “endoscopic”, “early gastric cancer”, “early esophageal cancer”, “early esophageal squamous cell carcinoma”, “early Barrett’s neoplasia”, “early esophageal adenocarcinoma”, “artificial intelligence”, “AI”, “machine learning”, “deep learning”, “artificial neural network”, “support vector machine”, “naive bayes”, and “classification tree”. Potentially relevant studies (based on title and abstract) were then read completely to ensure eligibility in the meta-analysis. In addition, we also reviewed the reference lists of relevant studies to search for eligible studies.

### Study Eligibility Criteria

Studies meeting the following criteria were included in the meta-analysis: (1) studies that evaluated AI diagnostic performance for EUGIC using endoscopic images; (2) true positive (TP), false positive (FP), false negative (FN), and true negative (TN) values could be extracted directly or calculated from the original studies. The following studies were excluded from our meta-analysis: (1) reviews, (2) meta-analyses, and (3) comments or protocols. We followed a strict exclusion policy that any study meeting one of the abovementioned exclusion criteria was excluded.

### Data Extraction

Two authors (MZJ and XDL) separately extracted data from the included studies, namely, author, publication year, study design, imaging type, AI model, sample size, TP, FP, FN, and TN. TP, FP, FN, and TN were extracted with the histology as the reference standard. Intramucosal carcinoma, T1 cancer, and Barrett’s esophagus (BE) with high-grade dysplasia were considered as positive. Normal tissue, BE without high-grade dysplasia, and non-cancerous lesions were considered as negative. The authors of the studies were contacted for missing information, if necessary. Discrepancies were decided through discussion.

### Methodological Quality Assessment

Two authors (XDL and XCL) evaluated the quality and potential bias risk of eligible studies based on the Quality Assessment of Diagnostic Accuracy Studies (QUADAS-2) (14). Disagreements were resolved through discussion. The QUADAS-2 tool was composed of four domains: “patient selection”, “index test”, “reference standard”, and “flow and timing”. In addition, the “patient selection”, “index test”, and “reference standard” were further tested for “applicability”. Each domain was then stratified into high, low, or unclear bias risk.

### Statistical Analysis

Statistical analysis was performed using the Meta-Disc software (version 14). To assess AI performance in EUGIC diagnosis, the pooled sensitivity, specificity, positive likelihood ratio (PLR), negative likelihood ratio (NLR), and diagnostic odds ratio (DOR) with 95% confidence interval (CI) were computed. In addition, we plotted a summary receiver operating characteristic (SROC) curve. The area under the curve (AUC) was computed to predict precision in diagnosis. We evaluated AI diagnostic performance based on images (image-based analysis) and patients (patient-based analysis). The forest plot was constructed. The inconsistency index (*I* ^2^) test determined presence or absence of heterogeneity among studies using sensitivity (15). A fixed-effects model was used if the *I* ^2^ value < 50%; otherwise, a random-effects model was selected. The Spearman correlation coefficient (SCC) between sensitivity and false positive rate was calculated, and a value > 0.6 indicated a threshold effect.

## Results

### Literature Screening and Bias Evaluation

**Figure 1:**
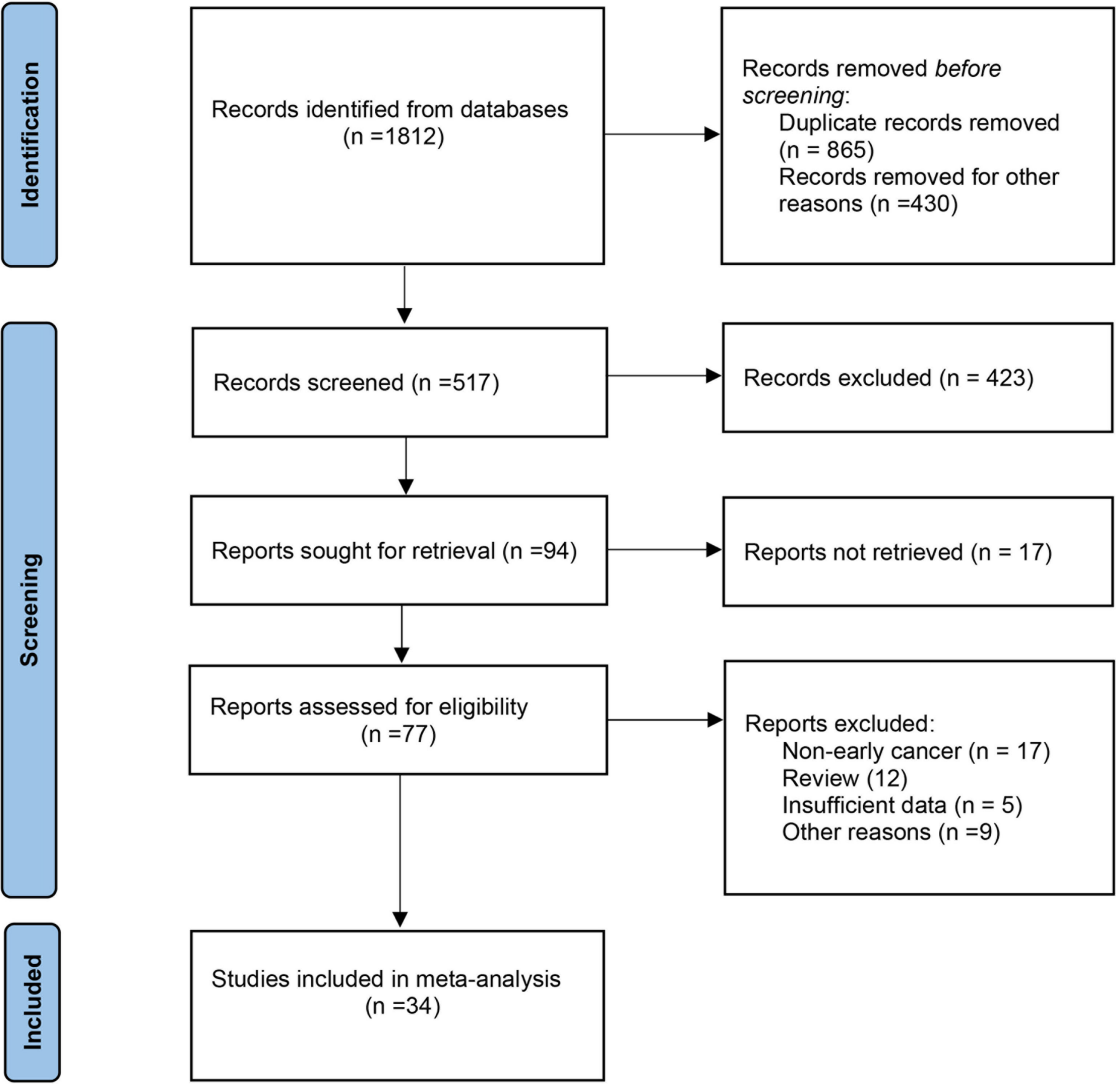
An overview of the study screening process.

**Figure 2:**
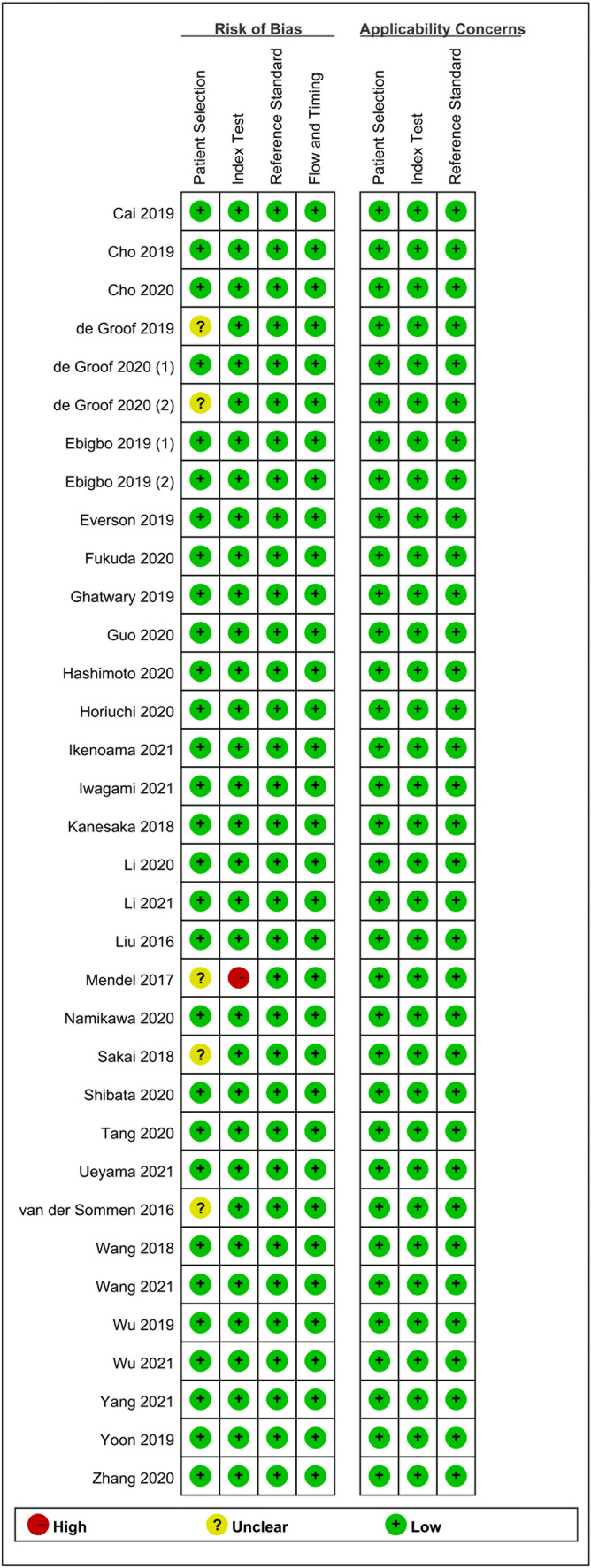
The quality assessment and risk of bias for each eligible study.

The primary screening uncovered 1,812 eligible studies. Upon removal of duplicates and other studies that were irrelevant to this study (based on title, abstract, and full article), 34 studies (16–49) investigating AI-assisted early esophageal cancer (EEC) and early gastric cancer (EGC) detection were included in the final meta-analysis. Among 34 studies, 18 and 17 studies assessed the diagnostic ability of AI in the detection of EEC (16–33) and EGC (29, 34–49), respectively. An overview of the eligible studies screening process is illustrated in **Figure 1**. **Table 1** presents the characteristics of all eligible studies. Overall, the included studies showed high methodological quality. The quality assessment and risk of bias for each eligible study are summarized in **Figure 2**.

**Table 1:** Clinical characteristics of the included studies.

Author/year	Study design	Imaging type	AI model	No. of images/patients/lesions in the test dataset		TP	FP	FN	TN	Endoscopist control
				Positive	Negative					
Cai, 2019 (16)	Retrospective	WLI	CNN	EESCC:91	Normal:96	89	14	2	82	Yes
de Groof, 2019 (17)	Prospective	WLI	CNN	EEAC:40/40^*^	BE:20/20^*^	38/38^*^	3/3^*^	2/2^*^	17/17^*^	No
de Groof, 2020 (1) (18)	Prospective	WLI	CNN	EEAC:33/10^*^	BE:111/10^*^	25/9^*^	15/1^*^	8/1^*^	96/9^*^	No
de Groof, 2020 (2) (19)	Prospective	WLI	CNN	EEAC:209	BE:248	186	31	23	217	No
Ebigbo, 2019 (1) (20)	Retrospective	WLI	CNN	EEAC:36	BE:26	30	0	6	26	No
Ebigbo, 2019 (2) (21)	Retrospective	WLI/NBI	CNN	EEAC:83^①^/33^②^ /33^①*^/33^②*^	BE:91^①^/41^②^ /41^①*^/41^②*^	78^①^/31^②^ /32^①*^/31^②*^	5^①^/8^②^ /5^①*^/8^②*^	5^①^/2^②^ /1^①*^/2^②*^	86^①/^33^②^ /36^①*^/33^②*^	No
Mendel, 2017 (22)	Prospective	WLI	CNN	EEAC:50/22^*^	BE:50/17^*^	47	6	3	44	No
Everson, 2019 (23)	Retrospective	NBI	CNN	EESCC:775/10^*^	Normal:891/7^*^	770	24	5	867	No
Fukuda, 2020 (24)	Retrospective	NBI	CNN	EESCC:45/45^*^	NC:49/99^*^	39/41^*^	5/48^*^	6/4^*^	44/51^*^	Yes
Ghatwary, 2019 (25)	Retrospective	WLI	SSD	EEAC:50/22^*^	BE:50/17^*^	48	4	2	46	No
Guo, 2020 (26)	Retrospective	NBI	CNN	EESCC:1,480	NC:5,191	1,451	258	29	4,933	No
Iwagami, 2021 (27)	Retrospective	WLI+NBI	CNN	EEAC:36^*^	NC:43^*^	34^*^	25^*^	2^*^	18^*^	No
Li, 2021 (28)	Retrospective	WLI/NBI	CNN	EESCC:133^①^/133^②^	Normal:183^①^/183^②^	131^①^/121^②^	31^①^/6^②^	2^①^/12^②^	152^①^/177^②^	Yes
Liu, 2016 (29)	Retrospective	WLI	SVM	EEC:150	Normal:250	140	27	10	233	No
Hashimoto, 2020 (30)	Retrospective	WLI/NBI	CNN	EEAC:146^①^/79^②^	BE:107^①^/126^②^	144^①^/73^②^	12^①^/1^②^	2^①^/6^②^	95^①^/125^②^	No
van der Sommen, 2016 (31)	Retrospective	WLI	SVM	EEAC:60/21^*^	BE:40/23^*^	50/18^*^	7/3^*^	10/3^*^	33/20^*^	Yes
Wang, 2021 (32)	Retrospective	WLI/NBI	CNN	EEAC:95^①^/115^②^	Normal:17^①^/37^②^	90^①^/112^②^	4^①^/12^②^	5^①^/3^②^	13^①^/25^②^	No
Yang, 2021 (33)	Retrospective	WLI	CNN	EESCC:474/98^*^	Normal:964/787^*^	419/94^*^	9/13^*^	55/4^*^	955/774^*^	No
Wang, 2018 (34)	Retrospective	WLI	CNN	EGC:232	NC + normal:478	206	49	26	429	Yes
Horiuchi, 2020 (35)	Retrospective	NBI	CNN	EGC:151	NC:107	144	31	7	76	No
Ikenoama, 2021 (36)	Retrospective	WLI	CNN	EGC:209	NC:2,731	122	347	87	2,384	Yes
Kanesaka, 2018 (37)	Retrospective	NBI	SVM	EGC:61	NC:20	59	1	2	19	No
Li, 2020 (38)	Retrospective	NBI	CNN	EGC:170	NC:171	155	16	15	155	No
Liu, 2016 (29)	Retrospective	WLI	SVM	EGC:130	Normal:270	118	25	12	245	No
Namikawa, 2020 (39)	Retrospective	WLI+NBI	CNN	EGC:100^*^	GU:120^*^	99^*^	8^*^	1^*^	112^*^	No
Shibata, 2020 (40)	Retrospective	WLI	CNN	EGC:533	Normal:1,208	404	127	129	1,081	No
Tang, 2020 (41)	Retrospective	WLI	CNN	EGC:4,810	NC:6,120	4,555	1,074	255	5,046	No
Ueyama, 2021 (42)	Retrospective	NBI	CNN	EGC:1,430	NC:870	1,401	0	29	870	No
Wu, 2021 (43)	Prospective	WLI	CNN	EGC:3^#^	NC:191^#^	3^#^	30^#^	0^#^	161^#^	No
Sakai, 2018 (44)	Retrospective	WLI	CNN	EGC:4,653	Normal:4,997	3,723	262	930	4,735	No
Yoon, 2019 (45)	Retrospective	WLI	CNN	EGC:330	NC:330	300	8	30	322	No
Wu, 2019 (46)	Retrospective	WLI	CNN	EGC:100	NC:100	94	9	6	91	No
Zhang, 2020 (47)	Retrospective	WLI	CNN	EGC:333	NC:311	285	189	48	122	No
Cho, 2019 (48)	Retrospective	WLI	CNN	EGC:46	NC:126	13	15	33	111	No
Cho, 2020 (49)	Retrospective	WLI	CNN	EGC:179	NC:217	111	75	68	142	No

EESCC, early esophageal squamous cell carcinoma; EEAC, early esophageal adenocarcinoma; BE, Barrett’s esophagus; EEC, early esophageal cancer; GU, gastric ulcers; SVM, support vector machine; CNN, convolutional neural network; SSD, single-shot multibox detector; WLI, white-light imaging; BNI, narrow-band imaging; NC, non-cancerous; TP, true positive; FP, false positive; FN, false negative; TN, true negative; WLI/NBI indicates that one study included WLI and BNI images, and the numbers of TP, FP, FN, and TN for EEC/EGC diagnosis with WLI or NBI images were reported or could be calculated; WLI + NBI indicates that one study included WLI and BNI images, but the numbers of TP, FP, FN, and TN for EEC/EGC diagnosis with WLI or NBI images were not reported or could not be calculated.

① indicates the number of WLI images; ② indicates the number of NBI images; *indicates the number of patients; ^#^indicates the number of lesions.

### AI-Assisted EEC Diagnosis Using Endoscopic Images

#### Meta-Analysis of AI-Assisted EEC Diagnosis Using Endoscopic Images [White-Light Imaging (WLI)/Narrow-Band Imaging (NBI) Images]

Eigtheen studies (16–33) reported the AI-assisted EEC diagnosis performance using endoscopic images. Moreover, 17 and 7 studies reported the AI-assisted EEC diagnosis performance based on per image (16–26, 28–33) and per patient (17, 18, 21, 24, 27, 31, 33), respectively. Among the 17 image-based studies, a total of 13,091 images (4,310 positive vs. 8,781 negative) were identified. Specifically, the positive group composed of the early esophageal squamous cell carcinoma (EESCC), early esophageal adenocarcinoma (EEAC), and EEC images, whereas the negative group consisted of normal, Barrett’s esophagus, and non-cancerous images. In most studies, the AI algorithm type was convolutional neural network (CNN). However, single-shot multibox detector (SSD) (25) and support vector machine (SVM) (29, 31) were also employed. Among the seven patient-based studies, a total of 1,380 patients (316 positive vs. 1,064 negative) were identified. Specifically, EESCC and EEAC constituted the positive group, whereas normal, Barrett’s esophagus, and non-cancerous comprised of the negative group. Most studies used the CNN algorithm. However, SVM was used in one study (31).

**Figure 3:**
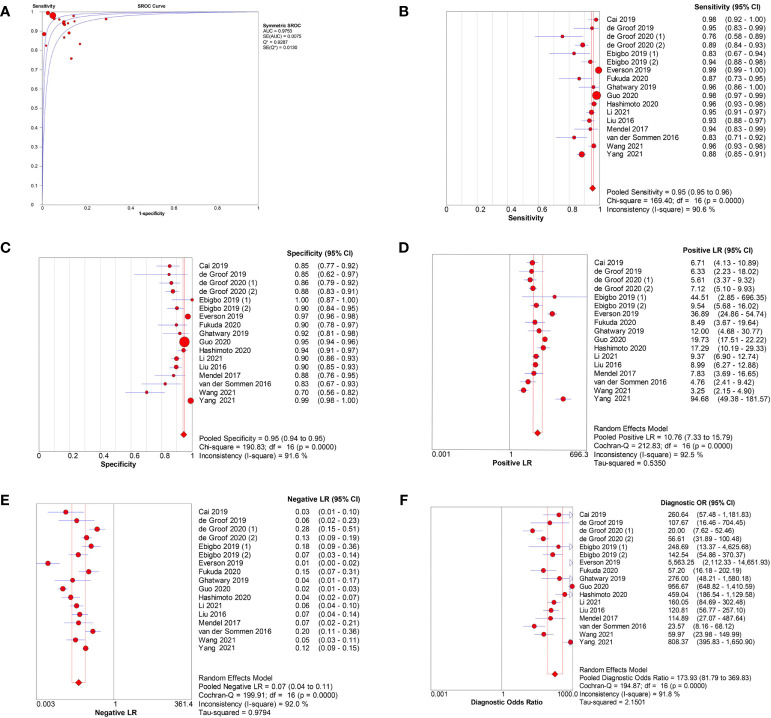
Meta-analysis of AI-assisted EEC diagnosis (image-based analysis). **(A)** SROC curve. **(B)** Pooled sensitivity. **(C)** Pooled specificity. **(D)** Pooled PLR. **(E)** Pooled NLR. **(F)** Pooled DOR.

In the 17 image-based studies investigating AI-assisted EEC diagnosis, the pooled AUC, sensitivity, specificity, PLR, NLR, and DOR were 0.98, 0.95 (95% CI, 0.95–0.96), 0.95 (95% CI, 0.94–0.95), 10.76 (95% CI, 7.33–15.79), 0.07 (95% CI, 0.04–0.11), and 173.93 (95% CI,81.79–369.83), respectively (**Figures 3A–F**). In addition, the SCC and p-values were −0.10 and 0.70 (>0.05), respectively, suggesting no significant threshold effect among these studies.

**Figure 4:**
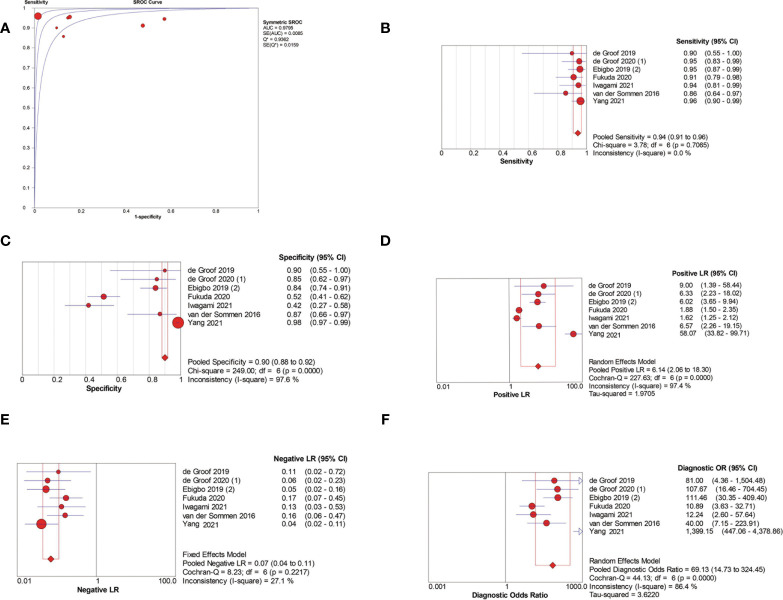
Meta-analysis of AI-assisted EEC diagnosis (patient-based analysis). **(A)** SROC curve. **(B)** Pooled sensitivity. **(C)** Pooled specificity. **(D)** Pooled PLR. **(E)** Pooled NLR. **(F)** Pooled DOR.

Among the seven patient-based studies investigating AI-assisted EEC diagnosis, the pooled AUC, sensitivity, specificity, PLR, NLR, and DOR were 0.98, 0.94 (95% CI, 0.91–0.96), 0.90 (95% CI, 0.88–0.92), 6.14(95% CI, 2.06–18.30), 0.07 (95% CI, 0.04–0.11), and 69.13 (95% CI, 14.73–324.45), respectively (**Figures 4A–F**). The SCC and p-values were −0.071 and 0.879 (>0.05), respectively, indicating no significant threshold effect among these studies.

### AI-Assisted EGC Diagnosis Using Endoscopy Images

#### Meta-Analysis of AI-Assisted EGC Diagnosis Using Endoscopic Images (WLI/NBI Images)

Seventeen studies (29, 34–49) reported the AI diagnosis performance of EGC using endoscopic images. Fifteen studies (29, 34–38, 40–42, 44–49), one study (39), and one study (43) evaluated the AI diagnosis performance based on per image, per patient, and per lesion, respectively.

Among the 15 image-based studies, a total of 31,423 images (13,367 positive vs. 18,056 negative) were identified. Only the EGC images were categorized in the positive group, whereas the normal and non-cancerous images were categorized in the negative group. A majority of the studies used CNN algorithm. However, the SVM algorithm was also used (29, 37). Among the two patient/lesion-based studies, a total of 414 patients/lesions (103 positive vs. 311 negative) were identified. Only the EGC were placed in the positive group, whereas the gastric ulcers and non-cancerous were placed in the negative group. Both studies utilized CNN algorithm.

**Figure 5:**
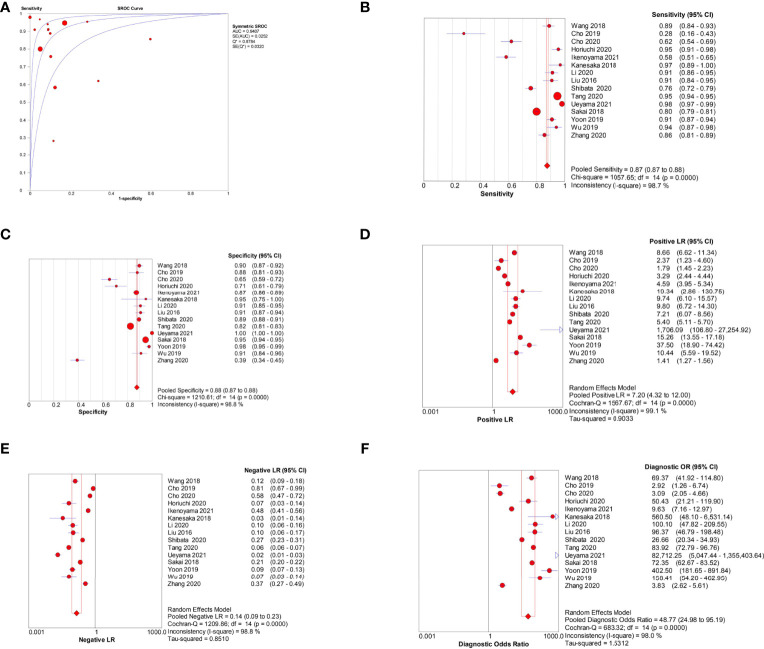
Meta-analysis of AI-assisted EGC diagnosis (image-based analysis). **(A)** SROC curve. **(B)** Pooled sensitivity. **(C)** Pooled specificity. **(D)** Pooled PLR. **(E)** Pooled NLR. **(F)** Pooled DOR.

Among the 15 image-based EGC detection studies, the pooled AUC, sensitivity, specificity, PLR, NLR, and DOR were 0.94, 0.87 (95% CI, 0.87–0.88), 0.88 (95% CI, 0.87–0.88), 7.20 (95% CI, 4.32–12.00), 0.14 (95% CI, 0.09–0.23), and 48.77 (95% CI, 24.98–95.19), respectively (**Figures 5A–F**). The SCC and p-values were −0.44 and 0.10 (>0.05), respectively, suggesting no significant threshold effect among these studies.

Only two patient-based studies evaluated AI in the diagnosis of EGC, so meta-analysis was not performed. In Namikawa’s study, the sensitivity and specificity were 0.99 and 0.93, respectively. In Wu’s study, the sensitivity and specificity were 1.00 and 0.8429, respectively.

### Subgroup Analysis Based on Imaging Type

To compare the AI diagnostic performance of EEC and EGC detection using WLI and NBI endoscopic images, we performed a subgroup analysis based on imaging type. On the basis of the results of subgroup analysis, the NBI mode showed a better diagnostic performance than the WLI mode. The results are summarized in **Table 2**.

**Table 2:** Summary of subgroup analysis based on imaging type.

Subgroup	Number of included studies	Sensitivity (95% CI)	Specificity (95% CI)	PLR (95% CI)	NLR (95% CI)	DOR (95% CI)	AUC
**EEC**
WLI							
image-based analysis	14	0.92 (0.90–0.93)	0.93 (0.91–0.94)	9.11 (6.04-13.75)	0.09 (0.06–0.13)	136.06 (67.20–275.49)	0.97
patient-based analysis	5	0.95 (0.92-0.98)	0.82 (0.74–0.88)	4.70 (3.32–6.65)	0.07 (0.04–0.12)	86.48 (39.04–191.57)	0.95
BNI							
image-based analysis	7	0.98 (0.97–0.98)	0.95 (0.95–0.96)	14.00 (6.71–29.20)	0.05 (0.02–0.11)	363.56 (108.47–1218.26)	0.99
**EGC**
WLI							
image-based analysis	11	0.86 (0.85–0.87)	0.87 (0.87–0.88)	6.12 (3.53–10.63)	0.21 (0.12–0.35)	29.92 (14.23–62.90)	0.92
NBI							
image-based analysis	4	0.97 (0.96–0.98)	0.96 (0.95–0.97)	25.92 (1.63–413.31)	0.05 (0.02–0.12)	523.76 (37.39–7336.36)	0.99

#### Meta-Analysis of AI-Assisted EGC Diagnosis Using WLI Endoscopic Images

Fourteen studies (16–22, 25, 28–33) reported the performance of AI-assisted EEC detection using WLI endoscopic images. Among the 14 image-based studies, the pooled AUC, sensitivity, specificity, PLR, NLR, and DOR were 0.97, 0.92 (95% CI, 0.90–0.93), 0.93 (95% CI, 0.91–0.94), 9.11 (95% CI, 6.04-13.75), 0.09 (95% CI, 0.06–0.13), and 136.06 (95% CI, 67.20–275.49), respectively. The SCC and p-values were 0.24 and 0.40 (>0.05), respectively, indicating no significant threshold effect among these studies.

Among the five patient-based studies (17, 18, 21, 31, 33), the pooled AUC, sensitivity, specificity, PLR, NLR, and DOR were 0.95, 0.95 (95% CI, 0.92–0.98), 0.82 (95% CI, 0.74–0.88), 4.7 (95% CI, 3.32–6.65), 0.07 (95% CI, 0.04–0.12), and 86.48 (95% CI, 39.04–191.57), respectively. The SCC and p-values were 0.5 and 0.39 (>0.05), respectively, indicating no significant threshold effect among these studies.

#### Meta-Analysis of AI-Assisted EEC Diagnosis Using NBI Endoscopic Images

Seven studies (21, 23, 24, 26, 28, 30, 32) reported the AI-assisted EEC detection performance using NBI endoscopic images. Among the seven image-based studies, the pooled AUC, sensitivity, specificity, PLR, NLR, and DOR were 0.99, 0.98 (95% CI, 0.97–0.98), 0.95 (95% CI, 0.95–0.96), 14.00 (95% CI, 6.71–29.20), 0.05 (95% CI, 0.02–0.11), and 363.56 (95% CI, 108.47–1218.26), respectively. The SCC and p-values were −0.04 and 0.94 (>0.05), respectively, indicating no significant threshold effect among these studies. Only two patient-based studies evaluated AI for the diagnosis of EEC, so meta-analysis was not performed. In the study by Ebigbo et al., (21) the sensitivity and specificity were 0.94 and 0.80, respectively. In the study by Fukuda et al. (24), the sensitivity and specificity were 0.91 and 0.52, respectively.

#### Meta-Analysis of AI-Assisted EGC Diagnosis Using WLI Endoscopic Images

Twelve studies (29, 34, 36, 40, 41, 43–49) reported the AI diagnosis performance of EGC detection using WLI endoscopic images. Eleven studies (29, 34, 36, 40, 41, 44–49) reported the AI diagnosis performance based on per image. In addition, only Wu’s study (43) reported the AI diagnosis performance based on per lesion. Among the 11 image-based EGC detection studies, the pooled AUC, sensitivity, specificity, PLR, NLR, and DOR were 0.92, 0.86 (95% CI, 0.85–0.87), 0.87 (95% CI, 0.87–0.88), 6.12 (95% CI, 3.53–10.63), 0.21 (95% CI, 0.12–0.35), and 29.92 (95% CI, 14.23–62.90). The SCC and p-values were −0.35 and 0.30 (>0.05), respectively, indicating no significant threshold effect among these studies.

#### Meta-Analysis of AI-Assisted EGC Diagnosis Using NBI Endoscopic Images

Four studies (35, 37, 38, 42) reported the AI diagnosis performance for EGC using endoscopic NBI images based on per image. In addition, no studies reported the AI diagnosis performance based on per lesion or per patient. Among the four image-based EGC detection studies, the pooled AUC, sensitivity, specificity, PLR, NLR, and DOR were 0.99, 0.97 (95% CI, 0.96–0.98), 0.96 (95% CI, 0.95–0.97), 25.92 (95% CI, 1.63–413.31), 0.05 (95% CI, 0.02–0.12), and 523.76 (95% CI, 37.39–7336.36), respectively. The SCC and p-values were −0.8 and 0.2 (>0.05), respectively, suggesting no significant threshold effect among these studies.

### Subgroup Analysis Based on Pathologic Type in Esophagus

We also performed a subgroup analysis between early esophageal squamous cell carcinoma (EESCC) and early esophageal adenocarcinoma (EEAC). On the basis of the results of subgroup analysis, AI showed a better diagnostic performance in EESCC than EEAC. The results are summarized in **Table 3**.

**Table 3:** Summary of subgroup analysis based on pathologic type.

Subgroup	Number of included studies	Sensitivity (95% CI)	Specificity (95% CI)	PLR (95% CI)	NLR (95% CI)	DOR (95% CI)	AUC
EESCC							
image-based analysis	6	0.96 (0.96–0.97)	0.95 (0.95–0.96)	18.21 (10.07–32.93)	0.04 (0.01–0.11)	491.74 (170.20–1420.71)	0.99
EEAC							
image-based analysis	10	0.93 (0.91–0.94)	0.89 (0.87–0.91)	7.41 (5.09–10.77)	0.10 (0.06–0.15)	87.66 (44.40–173.08)	0.96
patient-based analysis	5	0.94 (0.89-0.97)	0.75 (0.68–0.81)	4.76 (1.69–13.38)	0.09 (0.05–0.17)	51.94 (20.89–129.11)	0.96

#### Meta-Analysis of AI-Assisted EESCC Diagnosis Using Endoscopic Images (WLI/NBI Images)

Six studies (16, 23, 24, 26, 28, 33) reported the AI-assisted EESCC diagnosis performance using endoscopic images based on per image. Among the six image-based studies, the pooled AUC, sensitivity, specificity, PLR, NLR, and DOR were 0.99, 0.96 (95% CI, 0.96–0.97), 0.95 (95% CI, 0.95–0.96), 18.21 (95% CI, 10.07–32.93), 0.04 (95% CI, 0.01–0.11), and 491.74 (95% CI, 170.20–1420.71), respectively. The SCC and p-values were −0.20 and 0.70 (>0.05), respectively, indicating no significant threshold effect among these studies. Only two patient-based studies (24, 33) evaluated AI for the diagnosis of EESCC, so meta-analysis was not performed. In the study by Yang et al. (33), the sensitivity and specificity were 0.97 and 0.99, respectively. In the study by Fukuda et al. (24), the sensitivity and specificity were 0.91 and 0.52, respectively.

#### Meta-Analysis of AI-Assisted EEAC Diagnosis Using Endoscopic Images (WLI/NBI Images)

Ten studies (17–22, 25, 30–32) reported the AI-assisted EEAC diagnosis performance using endoscopic images based on per image. Among the 10 image-based studies, the pooled AUC, sensitivity, specificity, PLR, NLR, and DOR were 0.96, 0.93 (95% CI, 0.91–0.94), 0.89 (95% CI, 0.87–0.91), 7.41 (95% CI, 5.09–10.77), 0.10 (95% CI, 0.06–0.15), and 87.66 (95% CI, 44.40–173.08), respectively. The SCC and p-values were −0.03 and 0.93 (>0.05), respectively, indicating no significant threshold effect among these studies.

Among the five patient-based studies (17, 18, 21, 27, 31), the pooled AUC, sensitivity, specificity, PLR, NLR, and DOR were 0.96, 0.94 (95% CI, 0.89–0.97), 0.75 (95% CI, 0.68–0.81), 4.76 (95% CI, 1.69–13.38), 0.09 (95% CI, 0.05–0.17), and 51.94 (95% CI, 20.89–129.11), respectively. The SCC and p-values were 0.6 and 0.29 (>0.05), respectively, indicating no significant threshold effect among these studies.

## Discussion

In this study, we conducted a comprehensive literature search and included all studies that assessed diagnostic performance of AI in EUGIC using endoscopic images. Next, we conducted a meta-analysis to explore the diagnostic performance of AI in EUGIC detection. On the basis of our results, AI demonstrated an excellent diagnostic ability, with high accuracy, sensitivity, specificity, PLR, and DOR, and with low NLR in detecting EUGIC, suggesting the feasibility of AI-assisted EUGIC diagnosis in clinical practice. To the best of our knowledge, this is the first systematic review and meta-analysis that explored the AI-assisted detection of EUGIC based on upper gastrointestinal endoscopic images.

Endoscopy is the primary tool used in the diagnosis of UGIC (50, 51). However, EUGIC lesions manifest as indistinct mucosal alterations under the classic WLI images. Therefore, EUGIC detection is often highly dependent on endoscopist’s experience and expertise (52). Previous studies also revealed that WLI-based EGC diagnosis is possible, but with poor sensitivity or specificity (36, 47, 48). More recently, AI-assisted image recognition makes remarkable breakthroughs in the field of medical imaging diagnosis and is gaining popularity in clinical practice (7–11, 53, 54). Traditional AI algorithms like SVM and decision trees require experts to manually design the image features, before the algorithm extracts the feature from images (53, 55). This results in the detection of only specific lesions, and in case the features are insufficient, satisfactory identification results cannot be obtained. Simultaneously, manual design is highly dependent on the previous knowledge of designers. Thus, it is not feasible to work with large amounts of data. At present, many studies on medical image recognition adopt deep learning algorithm based on CNN. The deep learning can automatically learn the most predictive characteristics from a large image data file with no requirement of previous knowledge and classify these images. In our study, most included studies used the CNN algorithm, so we did not compare the AI diagnostic ability between the different algorithms. Many studies demonstrated excellent AI performance in detecting early esophageal and stomach cancers with the CNN algorithm. Consistent with these studies, in our study, AI exhibited an excellent diagnosis performance for EUGIC with high accuracy, sensitivity, and specificity.

Although several advanced technologies like NBI, confocal laser endomicroscopy, and blue laser imaging have shown great promise in the endoscopic detection of EUGIC, endoscopists still need extensive specialized training and substantial experience to identify early cancer lesions accurately. NBI endoscopy is an optical image-enhanced technology that better visualizes surface structures and blood vessels than does WLI (56). Multiple studies have demonstrated NBI has a high sensitivity in detecting EUGIC (37, 57, 58). To compare the AI diagnostic performance for EUGIC detection using WLI and NBI endoscopic images, we performed a subgroup analysis based on imaging type. On the basis of our results, the NBI imaging mode has a superior diagnostic performance for both EEC and EGC detection, with higher AUC, sensitivity, specificity, PLR and DOR, and lower NLR.

There are limitations to this study. First, most studies were based on the retrospective review of selected images. At the same time, the number of positive images and negative images included in some included studies was significantly different. All retrospective studies were considered at high risk for selection bias, so those studies might overestimate the diagnostic accuracy of AI. Second, few studies compared the diagnostic efficacy between AI and endoscopists, so we could not perform meta-analysis to compared the diagnostic efficacy between AI and endoscopists.

In conclusion, on the basis of our meta-analysis, AI achieved high accuracy in diagnosis of EUGIC. Further prospective studies comparing the diagnostic performance between AI and endoscopists are warranted.

## Data Availability Statement

The original contributions presented in the study are included in the article/supplementary material. Further inquiries can be directed to the corresponding author.

## Author Contribution

DL designed the study. FK and JD screened electronic databases. MZ and XDL extracted data from the selected articles. XDL and XCL evaluated eligible study quality and potential bias risk. Statistical analyses were performed by YT and BL. DL wrote the manuscript. SS supervised the study. All authors contributed to the article and approved the submitted version.

## Funding

This study is supported by the Key Research and Development Project of Science & Technology Department of Sichuan Province (20ZDYF1129) and the Applied Basic Research Project of Science & Technology Department of Luzhou city (2018-JYJ-45).

## Conflict of Interest

The authors declare that the research was conducted in the absence of any commercial or financial relationships that could be construed as a potential conflict of interest.

## Publisher’s Note

All claims expressed in this article are solely those of the authors and do not necessarily represent those of their affiliated organizations, or those of the publisher, the editors and the reviewers. Any product that may be evaluated in this article, or claim that may be made by its manufacturer, is not guaranteed or endorsed by the publisher.
